# Al-Biruni Earth Radius Optimization with Transfer Learning Based Histopathological Image Analysis for Lung and Colon Cancer Detection

**DOI:** 10.3390/cancers15133300

**Published:** 2023-06-23

**Authors:** Rayed AlGhamdi, Turky Omar Asar, Fatmah Y. Assiri, Rasha A. Mansouri, Mahmoud Ragab

**Affiliations:** 1 Information Technology Department, Faculty of Computing and Information Technology, King Abdulaziz University, Jeddah 21589, Saudi Arabia; 2 Department of Biology, College of Science and Arts at Alkamil, University of Jeddah, Jeddah, Saudi Arabia; 3 Department of Software Engineering, College of Computer Science and Engineering, University of Jeddah, Jeddah, Saudi Arabia; 4 Prince Sattam Bin Abdulaziz University, Al-Kharj 11942, Saudi Arabia; 5 Department of Biochemistry, Faculty of Sciences, King Abdulaziz University, Jeddah 21589, Saudi Arabia; 6 Mathematics Department, Faculty of Science, Al-Azhar University, Naser City 11884, Cairo, Egypt; 7 King Abdulaziz University-University of Oxford Centre for Artificial Intelligence in Precision Medicines, King Abdulaziz University, Jeddah 21589, Saudi Arabia

**Keywords:** lung and colon cancer, computer-aided diagnosis, transfer learning, parameter optimization, medical image analysis

## Abstract

**Simple Summary:**

Histopathological image analysis can be used for the detection of lung and colon cancer by the investigation of microscopic images of tissue samples. Since manual diagnosis takes a long time and is subjected to differing opinions of doctors, automated lung and colon cancer diagnosis becomes necessary. Therefore, the purpose of the study is to develop a transfer learning approach for lung and colon cancer detection on histopathological image analysis. It involves leveraging pre-trained model to analyze histopathological images. In addition, the proposed model uses improved ShuffleNet with deep convolutional recurrent neural network for feature extraction and classification, respectively. Besides, Al-Biruni Earth Radius Optimization and coati optimization algorithm are employed for hyperparameter tuning process. The experimental result analysis of the proposed model on the LC25000 database shows its promising performance on lung and colon cancer diagnosis.

**Abstract:**

An early diagnosis of lung and colon cancer (LCC) is critical for improved patient outcomes and effective treatment. Histopathological image (HSI) analysis has emerged as a robust tool for cancer diagnosis. HSI analysis for a LCC diagnosis includes the analysis and examination of tissue samples attained from the LCC to recognize lesions or cancerous cells. It has a significant role in the staging and diagnosis of this tumor, which aids in the prognosis and treatment planning, but a manual analysis of the image is subject to human error and is also time-consuming. Therefore, a computer-aided approach is needed for the detection of LCC using HSI. Transfer learning (TL) leverages pretrained deep learning (DL) algorithms that have been trained on a larger dataset for extracting related features from the HIS, which are then used for training a classifier for a tumor diagnosis. This manuscript offers the design of the Al-Biruni Earth Radius Optimization with Transfer Learning-based Histopathological Image Analysis for Lung and Colon Cancer Detection (BERTL-HIALCCD) technique. The purpose of the study is to detect LCC effectually in histopathological images. To execute this, the BERTL-HIALCCD method follows the concepts of computer vision (CV) and transfer learning for accurate LCC detection. When using the BERTL-HIALCCD technique, an improved ShuffleNet model is applied for the feature extraction process, and its hyperparameters are chosen by the BER system. For the effectual recognition of LCC, a deep convolutional recurrent neural network (DCRNN) model is applied. Finally, the coati optimization algorithm (COA) is exploited for the parameter choice of the DCRNN approach. For examining the efficacy of the BERTL-HIALCCD technique, a comprehensive group of experiments was conducted on a large dataset of histopathological images. The experimental outcomes demonstrate that the combination of AER and COA algorithms attain an improved performance in cancer detection over the compared models.

## 1. Introduction

Cancer can affect any organ of the human body; the foremost areas to be commonly affected are the brain, colon, skin, breasts, stomach, rectum, liver, prostate, and lungs. The common tumors to cause death in females and males are lung and colon cancer (LCC) [1]. When lung cells mutate uncontrollably, malignant cells appear, forming clusters called cancers. Globally, lung and colorectal (rectum and colon) tumors are the general kinds of tumors after breast cancer (BC) [2]. Moreover, colorectal and lung tumors have resulted in death rates of 9.4% and 18% correspondingly among all tumors. Hence, to explore the treatment options in the early phase of diseases, the precise detection of these tumor subtypes are essential. The noninvasive approaches for detection involve computed tomography (CT) imaging and radiography for flexible sigmoidoscopy and lung cancer and CT colonoscopy for colon tumors [3], but dependable classifying of these tumors is not probable utilizing noninvasive means at all times, and invasive processes such as histopathology are essential for accurate disease detection and the enhanced quality of treatments. As well, the manual grading of histopathologic images may be annoying for pathologists [4]. Likewise, the precise grading of the colon and lung tumor subtypes necessitates a trained pathologist, and manual grading can be prone to error. Now, automatic image processing techniques are being applied for lung tumors [5].

Artificial intelligence (AI) methods are utilized in the medical domain, such as the initial detection of biomedical images, health disasters, forecasts of diseases, etc. [6]. Deep learning (DL) methods can examine data in anatomical representations, high-dimensional images, and videos [7]. Likewise, DL methods derive hidden characteristics and features from healthcare images that are invisible to the naked eye for the initial cancer recognition and discrimination between its phases. DL refers to a subdivision of ML that eradicates the necessity for manual feature engineering, and CNN-related DL methods present hierarchical mapping features for superior representations of input images [8]. However, enormous data are needed for training large DL methods; transfer learning (TL) aids in adapting large pretraining methods for downstream tasks. Therefore, TL decreases the necessity for an enormous dataset for training that is rare in particular domains such as medicine [9]. TL and DL execute a crucial role in healthcare in framing automatic diagnostic mechanisms by utilizing healthcare images that include magnetic resonance images, histopathological images, radiographs, retina images, etc. Such automatic mechanisms are mainly utilized for classifier tasks and assist doctors in circumstances of automated quality checking and rapid data acquisition [10].

This manuscript offers the design of the Al-Biruni Earth Radius Optimization with Transfer Learning-based Histopathological Image Analysis for Lung and Colon Cancer Detection (BERTL-HIALCCD) technique. In the BERTL-HIALCCD technique, an improved ShuffleNet model is applied for the feature extraction process, and its hyperparameters are chosen by the BER algorithm. For the effectual detection of LCC, a deep convolutional recurrent neural network (DCRNN) model is applied. At the final stage, the coati optimization algorithm (COA) is exploited for the parameter selection of the DCRNN approach. The design of BER and COA for the hyperparameter tuning of the improved ShuffleNet and DCRNN models demonstrates the novelty of the work. To examine the result of the BERTL-HIALCCD technique, a comprehensive group of experiments was conducted on a large dataset of histopathological images.

## 2. Related Works

In [11], the authors utilized an AI-supported method and optimization approaches to realize the categorization of histopathologic images of colon and lung tumors. In the presented method, the image classes were trained with the DarkNet-19 technique, one of which was the DL method. With the equilibrium and Manta Ray Foraging optimizer methods, the selection of the ineffective attributes was attained. In the feature set mined from the DarkNet19 method, the potential attributes gained by the two utilized optimization methods were classified and integrated with the SVM approach. In [12], a hybrid classification method that included hog and daisy feature extraction modules and inception_v3 network were built to categorize lung tumors and normal tissues from lung pathological imagery. In [13], the authors presented a brief analysis of two feature extraction approaches for colon and lung tumor classification. In one method presented, six handcrafted extracted features methods dependent on shape, color, structure, and texture. The RF, Gradient Boosting (GB), MLP, and SVM-RBF methods with handcrafted attributes were tested and trained for lung and colon tumor categorization. In [14], the main intention of this case was to utilize digital histopathology images and a multi-input capsule network for framing an enhanced computerized diagnosis mechanism to find adenocarcinomas and squamous cell carcinomas of the lungs, in addition to adenocarcinomas of the colon. During the presented multi-input capsule network, two convolution layer blocks were utilized. The CLB (convolutional layer blocks) considered unprocessed histopathologic images as the input.

The authors of [15] presented a hybrid ensemble extracted feature method to proficiently find the LCC. It integrated ensemble learning and deep feature extraction with high-performance filters for cancer image data. Hamida et al. [16] concentrated on the usage of a DL structure for highlighting and classifying colon tumor regions in a sparsely annotated histopathologic data context. Firstly, the authors compared and reviewed the existing CNN that included the DenseNet, AlexNet, vgg, and ResNet Inception methods. The authors resorted to the use of TL methods to cope with the lack of a rich WSI dataset.

Mangal et al. [17] aimed to devise a computer-aided diagnosis system to find squamous cell carcinomas and adenocarcinomas of the lungs, in addition to adenocarcinomas of the colon, utilizing CNN by assessing the digital pathology images for these cancers. Ding et al. [18] designed FENet for genetic mutation estimation utilizing histopathologic images of colon cancer. Different to traditional methods of analyzing patch-related features alone without concerning their spatial connectivity, FENet incorporated feature enhancements in convolutional graph NN to combine discriminatory attributes with the capture gene mutation status.

In [19], a clinically comparable CNN structure-based approach to carry out an automated classifier of cancer grades and tissue infrastructures in hematoxylin and eosin-stained colon HSI was presented. It contained Enhanced Convolutional Learning Modules (ECLMs), a multi-level Attention Learning Module (ALM), and Transitional Modules (TMs). In [20], the efficiency of an extensive variety of DL-based structures was measured for the automatic tumor segment of colorectal tissue instances. The presented method demonstrated the efficacy of integrating CNN and TL in the encoded part of the segmentation structure for histopathology image diagnosis. In [21], the authors provided a systematic examination of XAI with an initial concentration on the methods that are presently being utilized in the domain of the healthcare system. In [22], the authors established a DL technique to predict disease-specific survival for stage II and III colorectal cancer utilizing 3652 cases. In [23], a novel approach dependent upon GCN for the early detection of COPD was presented that utilized lesser and weakly labeled chest CT image data in the openly accessible Danish Lung Cancer Screening Trial database. Jain et al. [24] demonstrated that lung cancer recognition depends on a histopathological image diagnosis utilizing DL structures. Afterwards, the image features were extracted utilizing Kernel PCA combined with CNN with KPCA (KPCA-CNN) utilized in the feature extracted layer of the CNN.

Although several LCC classification models are available in the literature, it is still required to enhance the detection performance. Most of the existing works did not focus on the hyperparameter optimization process. Generally, hyperparameter optimization helps in the identification of an optimal combination of hyperparameters for a given model architecture and dataset. It proficiently searches the hyperparameter space for the detection of the optimal configuration, which saves time and computational resources by automatically exploring different combinations rather than relying on manual trial-and-error approaches. It helps in quickly finding a good set of hyperparameters without exhaustively evaluating every possible combination. Therefore, in this work, the BER and COA are used for the hyperparameter tuning process.

## 3. Materials and Methods

**Figure 1:**
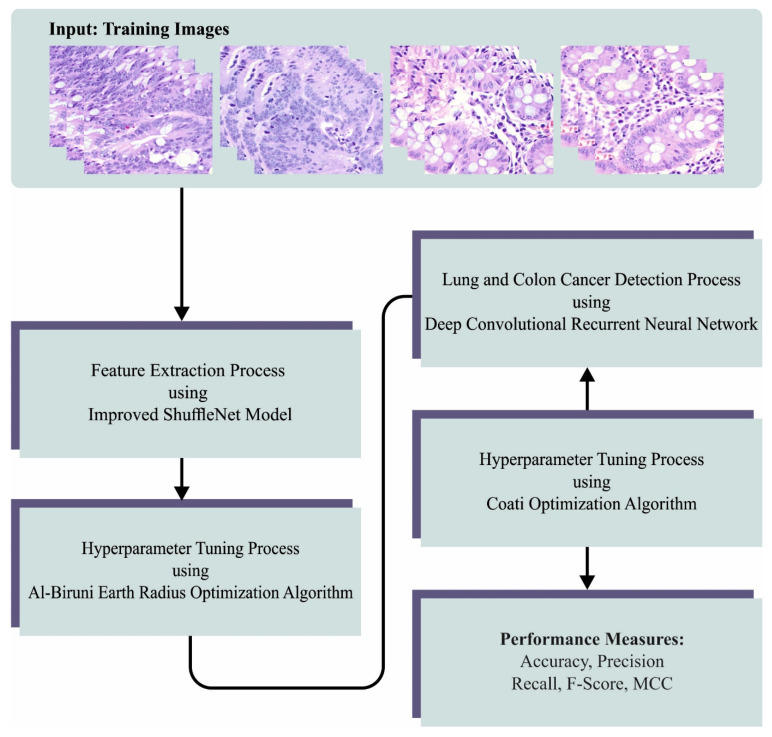
Overall flow of the BERTL-HIALCCD approach.

In this manuscript, we developed a novel LCC detection model named the BERTL-HIALCCD technique. The study aims to find LCC effectually on histopathological images. To achieve this, the BERTL-HIALCCD approach follows a series of subprocesses, namely improved ShuffleNet feature extraction, BER-based parameter optimization, DCRNN-based classification, and COA-based hyperparameter tuning. Figure 1 signifies the overall flow of the BERTL-HIALCCD system.

### 3.1. Dataset Used

The LC25000 dataset comprises 25,000 color images with 5 class labels of 5000 images each. The dataset is available at https://www.kaggle.com/datasets/andrewmvd/lung-and-colon-cancer-histopathological-images (accessed on 12 June 2023). Every image is 768 × 768 pixels and in .jpeg file format. The 5 class labels can be lung squamous cell carcinomas, colon adenocarcinomas, lung adenocarcinomas, and benign lung and benign colonic tissues.

### 3.2. Feature Extraction Using Improved ShuffleNet

In this work, the improved ShuffleNet method extracts features from histopathological images. Depthwise convolution (DW-Conv) is a special case of gathering convolutions in general and then evaluating a typical convolution with 1 × 1 dimensions for the depthwise convolutional procedure [25]:

(1) $$G_{i,j,m}=\overset{W,H}{\underset{w,h}{\sum }}K_{w,h,m}\cdot X_{i+w,j+h,m}$$

In Equation (1), G and K represent the resultant feature matrix and convolutional kernel weighted matrix, correspondingly. i, j, w, and h represents the coordinates of the related matrix. The depthwise separable convolution replaces the typical convolution, has lesser computations, and is typical for lightweight methods:

(2) $$Q_{1}=D_{f}^{2}D_{k}^{2}M+D_{f}^{2}M$$

(3) $$Q_{2}=D_{f}^{2}D_{k}^{2}MN$$

(4) $$\frac{Q_{1}}{Q_{2}}=\frac{D_{f}^{2}D_{k}^{2}M+D_{f}^{2}MN}{D_{f}^{2}D_{k}^{2}MN}=\frac{1}{N}+\frac{1}{D_{k}^{2}}$$

where $D_{f}$ and $D_{k}$ show the side size of the feature matrix and convolutional kernels, and $Q_{1}$ and $Q_{2}$ denote the computation count of depthwise convolutional and typical convolution correspondingly. M and N signify the number of channels in the output and input feature maps. Thus, the computation amount of the depthwise convolution is 1/9 of the typical convolution.

The channel shuffle method is a significant innovation point developed by ShuffleNet that realizes the data interchange of channels from the extracted feature method with smaller computation costs. In this work, effective channel attention (ECA) was established to suppress the relevant attributes and accomplish the weighting method of the features for succeeding the classifier models. However, when retaining the lightness, the LSR loss function can be performed when considering the multi-dimensional loss computation and enhances the model noise immunity.

The visual attention modules are drawn from human visual features to emphasize crucial data in images that are advantageous for enhancing the performance of the model. Visual attention mechanism brings accuracy improvements to CNNs by weighting the outcome features but mainly at the cost of enhancing the difficulty, namely convolution block attention mechanism (CBAM) SE. ECA refers to a lightweight attention model that borrowed the idea from SE to construct a channel attention module that is established in CNN to have participated during the end-to-end trained model. ECA exploits 1D convolutions for an extracted feature that prevents feature downscaling and efficiently captures cross-channel data interactions.

Assume the input feature matrix is $F\in {\mathbb{R}}^{C\times H\times W}$, and W, C, and H characterize the width, channels, and height of the input features, correspondingly. First, the input matrix can be processed via a global average pooling layer that leads to the channel feature description matrix $F\in {\mathbb{R}}^{C\times 1\times 1}$. Next, feature extraction can be executed utilizing a 1D convolutional layer, and the output can be processed via a nonlinear activation function:

(5) $$M_{c}\left(F\right)=\sigma \left(f_{1d}\left(F_{avg}\right)\right)$$

In Equation (5), σ represents the sigmoid activation function, and $f_{1d}$ designates the 1D convolutional process. Lastly, the input feature is multiplied with the attention weight for the channel size:

(6) $$F^{\prime }=F⊗M_{c}$$

In Equation (6), ⊗ represents elemental multiplication, $M_{c}$ is copies within the spatial size to attain C×H×W feature matrices that are later point multiplied with another matrix. The ECA belongs to the channel attention that enables the allocation of the weight to the channel of the feature maps, which makes the network focus on the most crucial channels. Thus, after this layer, the ECA module is embedded.

### 3.3. BER-Based Hyperparameter Optimization

For the effectual identification of the hyperparameter values of the improved ShuffleNet method, the BER algorithm is used. The optimizer technique aims to find the optimum solution for the problem with a set of constraints [26]. In this work, an individual from the population is characterized by the vector $\vec{S}=\left\{S_{1},\;S_{2},\;\ldots ,\;S_{d}\right\}\in R_{d}$, where d represents the searching space size, and $S_{j}$ indicates the feature or parameter from the optimizer problems. The fitness function f is used for determining how well an individual performs up to the provided point. The optimization process exploits the subsequent phase to search through the population for the certain optimal vector $S^{*}$, which increases the FF. This technique starts with a set of solutions. The following parameters are used for the optimization process: the population size, the FF, the dimension, and the lower and upper boundaries for the solution space.

In the proposed AER algorithm, the population is split into subgroups. The number of individuals in every group can be adapted to enhance the balance between the exploration and exploitation processes. Moreover, to assure the convergence of optimization techniques for the population, the elitism approach can be applied by keeping the leading solution when no best solution is found. If the fitness of the solution does not increase dramatically for 3 iterations, this is the local optima, and subsequently, other exploration individuals are produced by using the mutation process.

Exploration avoids local optimal stagnation by movement towards a better solution and is responsible for finding exciting locations in the search space.

Heading towards the best solution strategy is used to search for prospective regions around its existing location in the search space. This could be obtained by repetitively searching amongst near promising alternatives for a better option with respect to the fitness value:

(7) $$r=h\frac{cos\left(x\right)}{1-\cos\left(x\right)}$$

(8) $$\vec{D}=\vec{r_{1}}\cdot \left(\vec{S}\left(t\right)-1\right)$$

(9) $$\vec{S}\left(t+1\right)=\vec{S}\left(t\right)+\vec{D}\cdot \left(2\vec{r_{2}}-1\right)$$

where $\vec{D}$ denotes the diameter of the circle where the searching agent finds the potential area. If 0<x≤180, h denotes a random number within [0,2], and $\vec{r_{1}}$ and $\vec{r_{2}}$ show the coefficient vector which value was measured by $\vec{S}\left(t\right)$.

The exploitation team is responsible for improving the present solution. The BER evaluates every individual fitness value at all the cycles and differentiates the optimal individual. The BER applies two dissimilar strategies to accomplish exploitation, as follows.

The following equation is used for moving the searching agent towards the better solution:

(10) $$\vec{S}\left(t+1\right)=r^{2}\left(\vec{S}\left(t\right)+\vec{D}\right)$$

(11) $$\vec{D}=\vec{r_{3}}\left(\vec{L}\left(t\right)-\vec{S}\left(t\right)\right)$$

where $\vec{S}\left(t\right)$ indicates the solution vector at t iteration, $\vec{r_{3}}$ Denotes a random vector that controls the movement steps near the better solution, $\vec{D}$ denotes the distance vector, and $\vec{L}$ shows the better solution vector.

The most potential is the area surrounding the lead (better solution). Consequently, some individuals hunt in the surrounding area for a better solution with the possibility of finding the best solution. The BER exploits the subsequent formula to realize these operations.

(12) $$\vec{S}\left(t+1\right)=r\left(\vec{S*}\left(t\right)+\vec{k}\right)$$

(13) $$\vec{k}=z+\frac{2\times t^{2}}{N^{2}}$$

From the expression, $\vec{S*}$ represents the better solution, z denotes the random value within [0,1], t indicates the iteration value, and N indicates the overall number of iterations. 

A mutation is an alternative approach applied by the BER. It can be a genetic operator used for creating and sustaining population diversity. It avoids an earlier convergence by assisting in avoiding the local optima, such as a modification of the searching space as a springboard to other interesting topics. The mutation is crucial for the remarkable exploration of the BER.

(14) $$\vec{S}\left(t+1\right)=\vec{k}*z^{2}-h\frac{\cos\left(x\right)}{1-\cos\left(x\right)}$$

The BER selects the better performance for the following iteration to guarantee the quality of the defined solution. Even though the elitism technique improves the efficacy, it causes an early convergence when using a multi-modal function. Note that the BER gives impressive exploration abilities by applying a mutation method and searching nearby individuals to the exploration group. Due to its strong exploration abilities, the BER could prevent early convergence.

### 3.4. Detection Using Optimal DCRNN Model

At this stage, the features are passed to the DCRNN model for classification. Recently, a CNN was investigated and proved efficient in high-dimensional and large-scale data learning [27]. Additionally, a RNN is robust in long-term dependency capturing and temporal sequence learning. Here, the CNNs and RNNs are combined to implement feature learning on a MFCC-based representation depending on heart sounds that exploit the long-term dependency captured by the RNN and the encoded local features extracted from the CNN. The learnable kernel size in all the layers is fixed to 3 × 3, and the renowned ReLU function is exploited in all the convolution layers. A max-pooling is employed, followed by each convolutional layer, where 2 × 2 windows are exploited, and the stride is 2 × 2.

**Figure 2:**
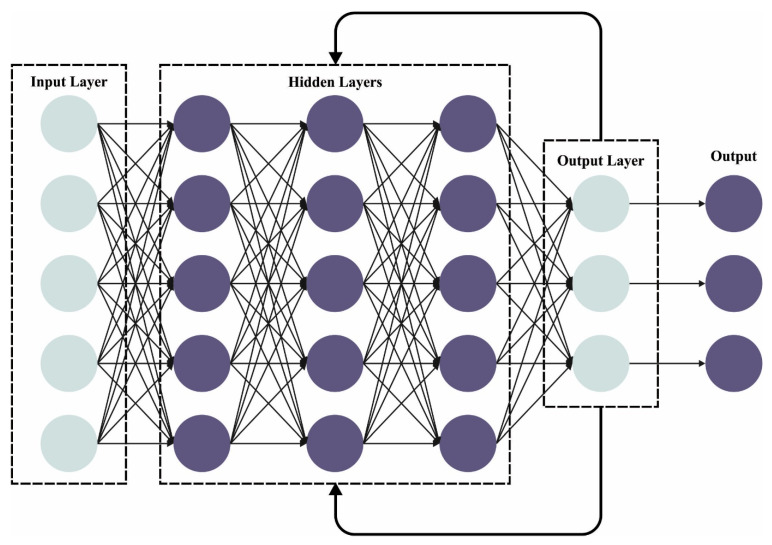
Framework of the CRNN.

The BN layer standardizes the mini-batch via the whole network, which reduces the internal covariate shift caused by progressive transform, and the dropout layer might prevent overfitting and decrease the amount of neurons. Therefore, for all the input samples, a feature map is attained. After the max-pooling and convolutional layers, an LSTM layer is exploited for learning the temporal characteristics amongst the attained mapping features, and an FC layer with sixty-four neurons is effectuated to learn the global features. Eventually, a softmax layer is executed for deriving the probability distribution through two classes respective to normal and abnormal heart sound signals. Figure 2 depicts the infrastructure of the CRNN.

To improve the detection rate, the COA is used for hyperparameter tuning. The updating method of a candidate solution (coatis place) from the COA dependent upon modeling 2 basic performances of coatis are given below [28]:

(i) Coatis’ escape strategy from the predator.
(ii) Coatis’ strategy while attacking iguanas.

Consequently, the COA population was upgraded 2 stages.

The primary stage of updating the coatis’ population is modeled by simulating the strategy while attacking the iguanas. Here, a set of coatis climb trees to attain an iguana and scare it. This approach leads the coatis to move toward dissimilar locations in the searching space, which illustrates their exploration capability in a global searching space. Here, the location of the fittest member of the population is considered the location of the iguana. Thus, the rise in the coatis’ location in the tree can be a mathematical formula:

(15) $$X_{i}^{P1}:x_{i,j}^{P1}=x_{i,j}+r\cdot \left(Iguana_{j}-I\cdot x_{i,j}\right),\;for\;i=1,\;2,\;\ldots ,\;\lfloor \frac{N}{2}\rfloor \;\mathrm{and}\;j=1,\;2,\;\ldots ,\;m.$$

Once the iguana falls to the ground, it can be positioned at an arbitrary location from the searching space. As per the arbitrary location, the coatis on the ground moves from the searching space, which can be given as follows:

(16) $$Iguana^{G}:Iguana_{j}^{G}=lb_{j}+r\cdot_{G}\left(ub_{j}.-.Ib_{j}\right),\;j=1,\;2,\;\ldots ,\;m,$$

(17) $$X_{i}^{P1}I:x_{i,j}^{P1}=\{\begin{array}{c} x_{i,j}+r\cdot (Iguona_{j}^{G}-I\cdot x_{i,j}),\;F_{Iguana^{G}}<F_{i},\\ x_{i,j}+r\cdot \left(x_{i,j}-Iguono_{j}^{G}\right),else \end{array}$$

$$for\;i=\lfloor \frac{N}{2}\rfloor +1,\lfloor \frac{N}{2}\rfloor +2,\ldots ,N\;and\;j=1,\;2,\;\ldots ,\;m$$

The new location evaluated for all the coatis is acceptable for the updating procedure once it enhances the value of the main function or the coatis remain in the prior location.

(18) $$X_{i}=\{\begin{array}{cc} X_{i}^{P1}, & F_{i}^{P1}<F_{i},\\ X_{i}, & else. \end{array}$$

Now, r represents a random real number within [0,1], $X_{i}^{P1}$ denotes the new location evaluated for the $i^{\mathrm{th}}$ coatis, $x_{i,j}^{P1}$ indicates the $j^{\mathrm{th}}$ dimension, $F_{i}^{P1}$ shows the objective function value, Iguana shows the iguana’s location in the search space that represents the location of the fittest member, $Igu{\mathrm{ana}}_{j}$ refers to the $j^{\mathrm{th}}$ dimension, $Iguana^{G}$ shows the location of the iguana on the ground that is produced at random, I $Iguana_{j}^{G}$ stands for the $j^{\mathrm{th}}$ dimension, ⌊·⌋ shows the floor function, and $F_{Iguana^{G}}$ denotes the value of the main function.

The second stage is to update the location of the coatis from the searching space using a mathematical process according to the natural behaviors of the coatis while encountering predators and escaping from them. Once the predator attacks a coati, the animal escapes during the locating. The coatis’ moves during these strategies result in a safer location closer towards the existing location that represents the exploitation capability during a local search.

To simulate these behaviors, a random location is produced nearby the location where every coati is located as follows:

(19) $$lb_{j}^{local}=\frac{lb_{j}}{t},ub_{j}^{local}=\frac{ub_{j}}{t},where\;t=1,\;2,\;\ldots ,\;T.$$

(20) $$X_{i}^{P2}\;:\;x_{i,j}^{P2}=x_{i,j}+\left(1-2r\right)\cdot \left(lb_{j}^{local}+r\cdot \left(ub_{j}^{local}-lb_{j}^{local}\right)\right),$$

i=1, 2, …, N, j=1, 2, …, m,

A recently estimated location is suitable once it enhances the value of the main function as follows:

(21) $$X_{i}=\{\begin{array}{cc} X_{i}^{P2}, & F_{i}^{P2}<F_{i},\\ X_{i}, & else, \end{array}$$

In Equation (21), $X_{i}^{P2}$ denotes the newest location evaluated for the $i^{\mathrm{th}}$ coatis, $x_{i,j}^{P2}$ shows the $j^{\mathrm{th}}$ dimension, $F_{i}^{P2}$ indicates the objective function values, r refers to the randomly generated values within [0,1], t shows the iteration counter, $lb^{local}$ and $ub^{local}$ denote the local lower, as well as upper, boundaries of the $j^{\mathrm{th}}$ search space correspondingly, and $lb_{j}$ and $ub_{j}$ indicate the lower boundaries, as well as upper boundaries, of the $j^{\mathrm{th}}$ search space, respectively.

The COA iteration is completed after the location of the coatis when the decision variable is updated. The updating process is based on Equations (15)–(21) and reiterated until it attains the maximum iteration. 

The COA method uses a fitness function (FF) to obtain a superior efficiency of the classifier. It describes a positive integer to suggest the finest outcome of the candidate solutions. The decline of the classifier rate of errors is assumed in the FF.

(22) $$fitness\left(x_{i}\right)=ClassifierErrorRate\left(x_{i}\right)\;=\frac{no.\;of\;misclassified\;instances\;}{Total\;no.\;of\;instances}*100$$

## 4. Performance Validation

**Figure 3:**
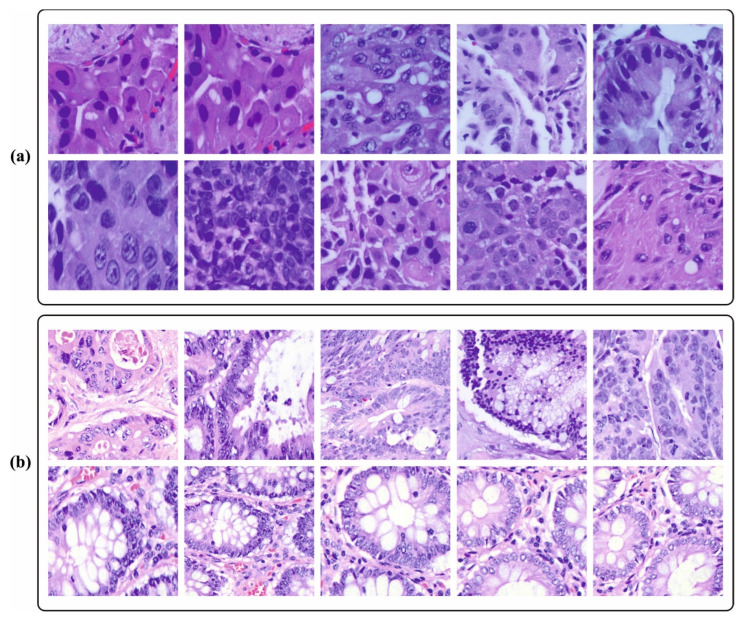
Sample images. (**a**) Lung cancer (**b**) Colon cancer.

In this section, the cancer detection outcomes of the BERTL-HIALCC technique are validated using the LC25000 database [29]. The database contains 25,000 instances with five classes, as provided in Table 1. Figure 3 demonstrates the sample images.

**Figure 4:**
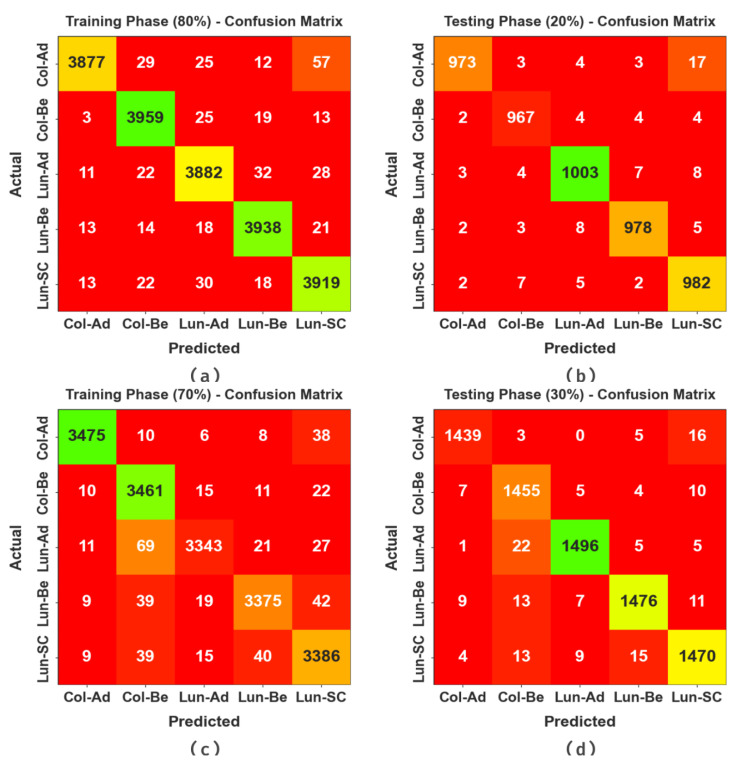
Confusion matrices of the BERTL-HIALCC system: (**a**,**b**) 80:20 of TRP/TSP and (**c**,**d**) 70:30 of TRP/TSP.

The confusion matrices of the BERTL-HIALCC method in the LCC detection results are depicted in Figure 4. The results identified that the BERTL-HIALCC technique recognized lung and colon cancers effectually.

**Figure 5:**
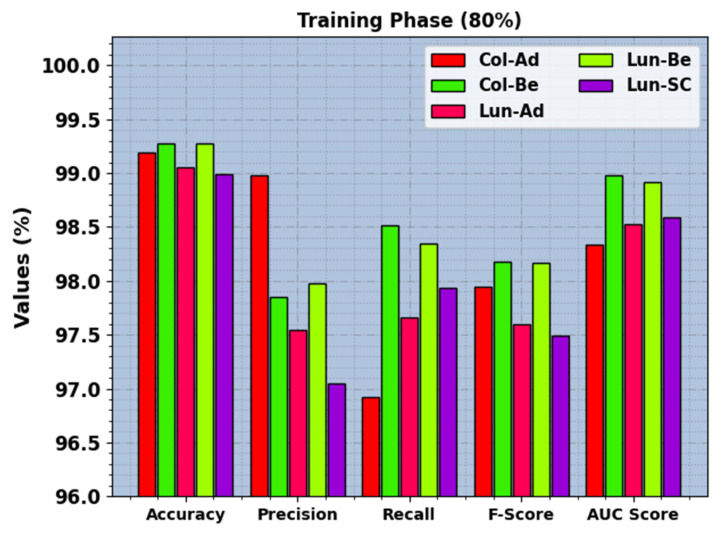
LCC recognition outcomes of the BERTL-HIALCC system on 70% of TRP.

In Table 2, the LCC recognition outcomes of the BERTL-HIALCC method under 80:20 of TRP/TSP are reported. In Figure 5, the recognition outcomes of the BERTL-HIALCC method are investigated under 80% of TRP. The figure indicates that the BERTL-HIALCC system identified the five classes proficiently. In the Col-Ad class, the BERTL-HIALCC technique provides $accu_{y}$, $prec_{n}$, $reca_{l}$, $F_{score}$, and $AUC_{score}$ of 99.19%, 98.98%, 96.92%, 97.94%, and 98.34%, respectively. Likewise, in the Col-Be class, the BERTL-HIALCC technique provides $accu_{y}$, $prec_{n}$, $reca_{l}$, $F_{score}$, and $AUC_{score}$ of 99.27%, 97.85%, 98.51%, 98.18%, and 98.98%, respectively. Similarly, in the Lun-SC class, the BERTL-HIALCC method provides $accu_{y}$, $prec_{n}$, $reca_{l}$, $F_{score}$, and $AUC_{score}$ of 98.99%, 97.05%, 97.93%, 97.49%, and 98.59%, correspondingly.

**Figure 6:**
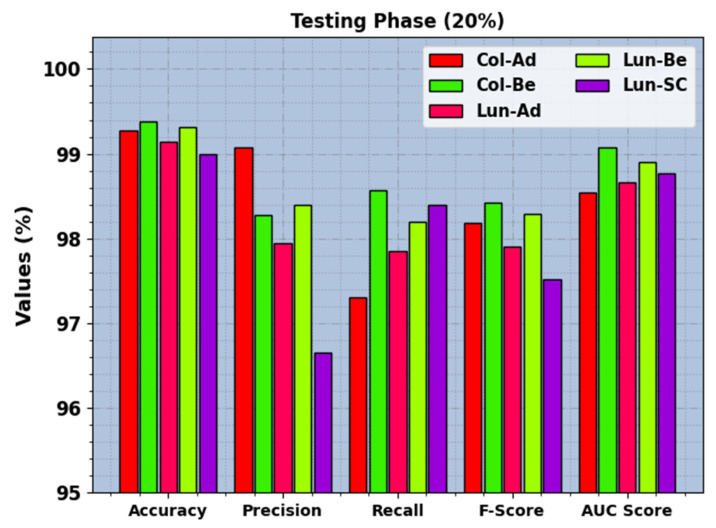
LCC recognition outcomes of the BERTL-HIALCC system on 30% of TSP.

In Figure 6, the recognition results of the BERTL-HIALCC technique are investigated under 20% of TRP. The figure revealed that the BERTL-HIALCC technique identified the five classes proficiently. In the Col-Ad class, the BERTL-HIALCC approach provided $accu_{y}$, $prec_{n}$, $reca_{l}$, $F_{score}$, and $AUC_{score}$ of 99.28%, 99.08%, 97.30%, 98.18%, and 98.54%, correspondingly. Similarly, in the Col-Be class, the BERTL-HIALCC method provided $accu_{y}$, $prec_{n}$, $reca_{l}$, $F_{score}$, and $AUC_{score}$ of 99.38%, 98.27%, 98.57%, 98.42%, and 99.07%, correspondingly. Similarly, in the Lun-SC class, the BERTL-HIALCC algorithm provides $accu_{y}$, $prec_{n}$, $reca_{l}$, $F_{score}$, and $AUC_{score}$ of 99%, 96.65%, 98.40%, 97.52%, and 98.77%, respectively.

**Figure 7:**
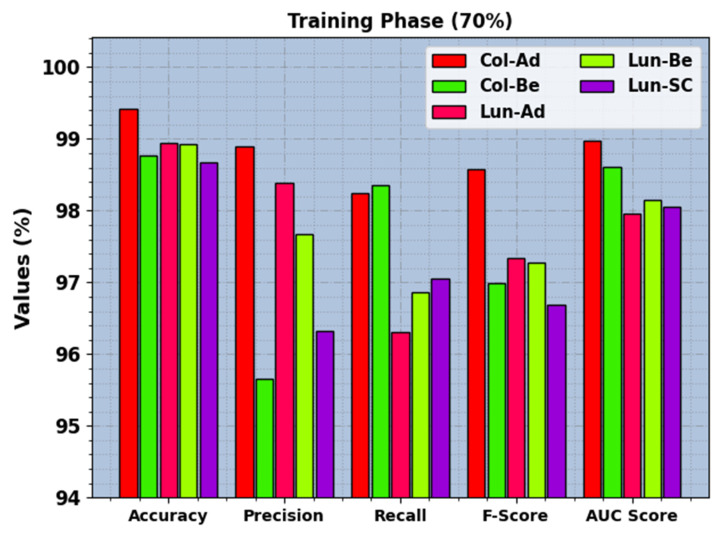
LCC recognition outcomes of the BERTL-HIALCC system on 80% of TRP.

In Table 3, the LCC recognition results of the BERTL-HIALCC method under 80:20 of TRP/TSP are reported. In Figure 7, the recognition outcomes of the BERTL-HIALCC method are investigated under 80% of TRP. The results point out that the BERTL-HIALCC system identified the five classes proficiently. In the Col-Ad class, the BERTL-HIALCC technique provides $accu_{y}$, $prec_{n}$, $reca_{l}$, $F_{score}$, and $AUC_{score}$ of 99.42%, 98.98%, 98.25%, 98.57%, and 98.98%, respectively. Likewise, in the Col-Be class, the BERTL-HIALCC approach offers $accu_{y}$, $prec_{n}$, $reca_{l}$, $F_{score}$, and $AUC_{score}$ of 98.77%, 95.66%, 98.35%, 96.99%, and 98.61%, correspondingly. Additionally, in the Lun-SC class, the BERTL-HIALCC technique provides $accu_{y}$, $prec_{n}$, $reca_{l}$, $F_{score}$, and $AUC_{score}$ of 98.67%, 96.33%, 97.05%, 96.69%, and 98.06%, correspondingly.

**Figure 8:**
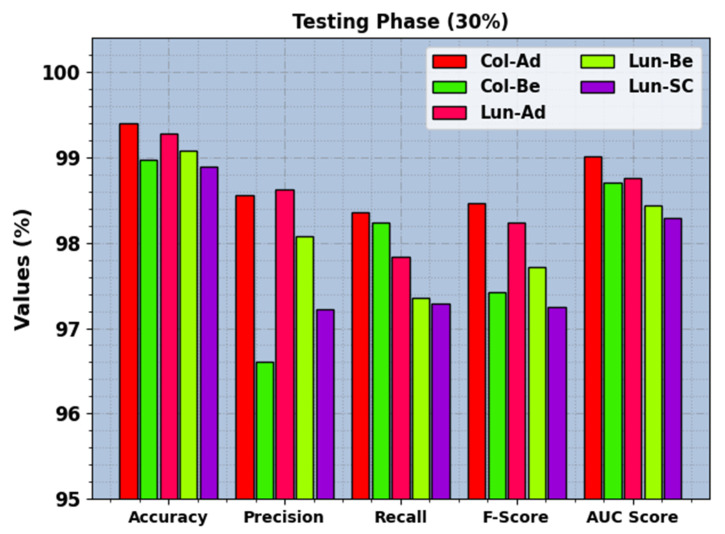
LCC recognition outcomes of the BERTL-HIALCC system on 30% of TSP.

In Figure 8, the recognition results of the BERTL-HIALCC technique are inspected under 20% of TRP. The results demonstrate that the BERTL-HIALCC technique identified the five classes proficiently. In the Col-Ad class, the BERTL-HIALCC technique provides $accu_{y}$, $prec_{n}$, $reca_{l}$, $F_{score}$, and $AUC_{score}$ of 99.40%, 98.56%, 98.36%, 98.46%, and 99.01%, respectively. Additionally, in the Col-Be class, the BERTL-HIALCC method offers $accu_{y}$, $prec_{n}$, $reca_{l}$, $F_{score}$, and $AUC_{score}$ of 98.97%, 96.61%, 98.24%, 97.42%, and 98.70%, respectively. Likewise, in the Lun-SC class, the BERTL-HIALCC approach presents $accu_{y}$, $prec_{n}$, $reca_{l}$, $F_{score}$, and $AUC_{score}$ of 98.89%, 97.22%, 97.29%, 97.25%, and 98.29%, respectively.

**Figure 9:**
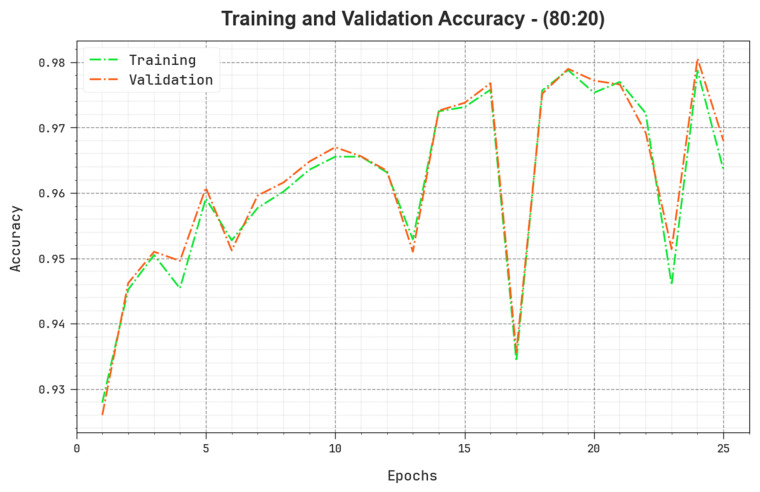
Accuracy curve of the BERTL-HIALCC system on 80:20 of TRP/TSP.

Figure 9 inspects the accuracy of the BERTL-HIALCC method in the training and validation on 80:20 of TRP/TSP. The figure specifies that the BERTL-HIALCC approach reaches greater accuracy values with higher epochs. In addition, the greater validation accuracy over training accuracy shows that the BERTL-HIALCC method learns productively on 80:20 of TRP/TSP.

**Figure 10:**
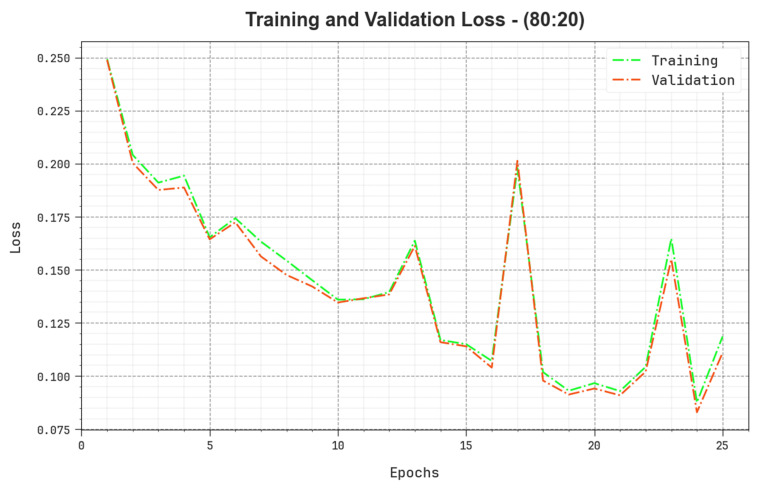
Loss curve of the BERTL-HIALCC system on 80:20 of TRP/TSP.

The loss analysis of the BERTL-HIALCC algorithm at the time of training and validation is validated on 80:20 of TRP/TSP in Figure 10. The results point out that the BERTL-HIALCC technique reaches similar values for the training and validation loss. The BERTL-HIALCC method learns productively on 80:20 of TRP/TSP.

**Figure 11:**
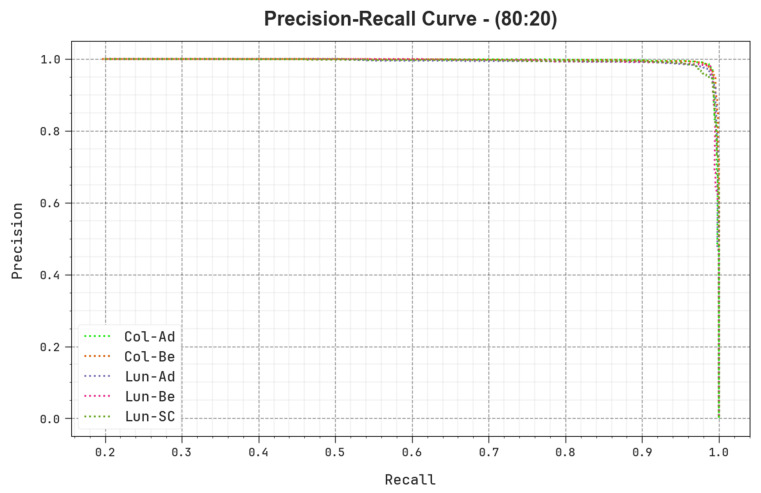
PR curve of the BERTL-HIALCC system on 80:20 of TRP/TSP.

A detailed precision–recall (PR) curve of the BERTL-HIALCC method is shown on 80:20 of TRP/TSP in Figure 11. The results show that the BERTL-HIALCC method results in increasing values of PR. In addition, the BERTL-HIALCC approach reached higher PR values in all the classes.

**Figure 12:**
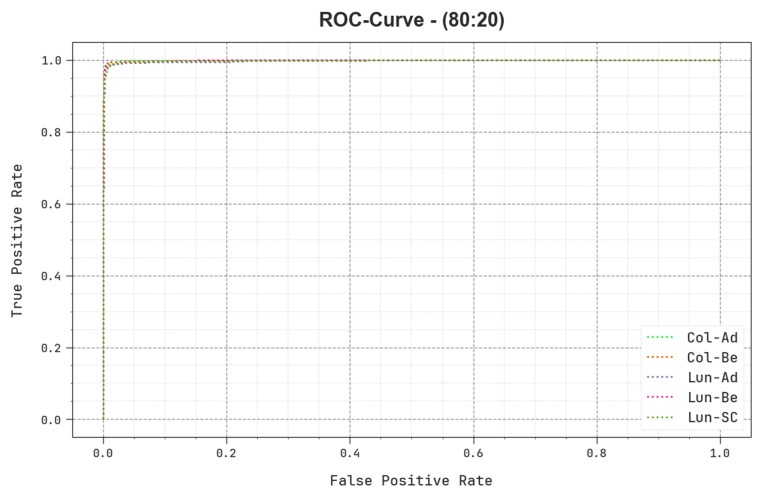
ROC curve of the BERTL-HIALCC system on 80:20 of TRP/TSP.

In Figure 12, a ROC study of the BERTL-HIALCC technique is revealed on 80:20 of TRP/TSP. The figure highlights that the BERTL-HIALCC method results in improved ROC values. In addition, the BERTL-HIALCC algorithm extends enhanced ROC values in all the classes.

To illustrate the improved cancer recognition results of the BERTL-HIALCC technique, a brief comparison study is carried out in Table 4 [30]. The results point out that the BERTL-HIALCC technique has improved results. Based on $accu_{y}$, the BERTL-HIALCC technique gains increasing $accu_{y}$ of 99.22%, while the MPADL-LC3, mSRC, Faster R-CNN, DAELGNN, ResNet50, CNN, and DL models accomplish decreasing $accu_{y}$ of 99.09%, 88.21%, 98.79%, 98.73%, 93.64%, and 97.11%, respectively.

Additionally, based on $prec_{n}$, the BERTL-HIALCC technique has an increasing $prec_{n}$ of 98.07%, while the MPADL-LC3, mSRC, Faster R-CNN, DAELGNN, ResNet50, CNN, and DL algorithms accomplish decreasing $prec_{n}$ of 98.01%, 85.21%, 96.53%, 97.95%, 96.12%, and 97.07%, respectively. Last, based on $reca_{l}$, the BERTL-HIALCC method has an increasing $reca_{l}$ of 98.06%, while the MPADL-LC3, mSRC, Faster R-CNN, DAELGNN, ResNet50, CNN, and DL approaches accomplish decreasing $reca_{l}$ of 97.20%, 91.78%, 97.78%, 97.63%, 96.39%, and 96.44%, correspondingly. In addition, the computation time (CT) analysis reported that the BERT-HIALCC technique results in the minimal CT value compared to the other models. Therefore, the proposed model can be employed for accurate LCC detection and classification.

## 5. Conclusions

In this study, we developed a novel LCC detection model named the BERTL-HIALCCD technique. This study aimed to identify LCC effectually on HSI. To achieve this, the BERTL-HIALCCD method followed a series of subprocesses, namely improved ShuffleNet feature extraction, BER-based parameter optimization, DCRNN-based classification, and COA-based hyperparameter tuning. Finally, a COA-based parameter selection process was carried out for the DCRNN model. To examine the performance of the BERTL-HIALCCD technique, a detailed set of experiments was conducted on a large dataset of HSI. The experimental results demonstrated that the combination of AER and COA algorithms attained an improved performance for cancer detection compared to the other models. In the future, the performance of the BERTL-HIALCDD method can be improved with multimodal fusion approaches. In addition, a histopathological image analysis can be integrated with clinical data, such as patient demographics, medical history, and genetic information, which can offer a better holistic understanding of cancer detection. It can significantly enhance the accuracy and reliability of the detection system and enable personalized medicine approaches. In addition, methods such as attention mechanisms, saliency maps, or feature visualization can assist in the identification of the important regions in histopathological images and provide insights into the decision-making process.

## Figures and Tables

**Table 1:** Details of the database.

Class Name	Description	No. of Instances
Col-Ad	Colon Adenocarcinoma	5000
Col-Be	Colon Benign Tissue	5000
Lun-Ad	Lung Adenocarcinoma	5000
Lun-Be	Lung Benign Tissue	5000
Lun-SC	Lung Squamous Cell Carcinoma	5000
Total No. of Samples		25,000

**Table 2:** LCC recognition outcomes of the BERTL-HIALCC system on 80:20 of TRP/TSP.

Class	$Accu_{y}$	$Prec_{n}$	$Reca_{l}$	$F_{score}$	$AUC_{score}$
Training Phase (80%)					
Col-Ad	99.19	98.98	96.92	97.94	98.34
Col-Be	99.27	97.85	98.51	98.18	98.98
Lun-Ad	99.05	97.54	97.66	97.60	98.52
Lun-Be	99.27	97.98	98.35	98.17	98.92
Lun-SC	98.99	97.05	97.93	97.49	98.59
Average	99.15	97.88	97.87	97.87	98.67
Testing Phase (20%)					
Col-Ad	99.28	99.08	97.30	98.18	98.54
Col-Be	99.38	98.27	98.57	98.42	99.07
Lun-Ad	99.14	97.95	97.85	97.90	98.66
Lun-Be	99.32	98.39	98.19	98.29	98.90
Lun-SC	99.00	96.65	98.40	97.52	98.77
Average	99.22	98.07	98.06	98.06	98.79

**Table 3:** LCC recognition outcomes of the BERTL-HIALCC system on 70:30 of TRP/TSP.

Class	$Accu_{y}$	$Prec_{n}$	$Reca_{l}$	$F_{score}$	$AUC_{score}$
Training Phase (70%)					
Col-Ad	99.42	98.89	98.25	98.57	98.98
Col-Be	98.77	95.66	98.35	96.99	98.61
Lun-Ad	98.95	98.38	96.31	97.34	97.96
Lun-Be	98.92	97.68	96.87	97.28	98.15
Lun-SC	98.67	96.33	97.05	96.69	98.06
Average	98.95	97.39	97.37	97.37	98.35
Testing Phase (30%)					
Col-Ad	99.40	98.56	98.36	98.46	99.01
Col-Be	98.97	96.61	98.24	97.42	98.70
Lun-Ad	99.28	98.62	97.84	98.23	98.75
Lun-Be	99.08	98.07	97.36	97.72	98.44
Lun-SC	98.89	97.22	97.29	97.25	98.29
Average	99.13	97.82	97.82	97.82	98.64

**Table 4:** Comparative outcomes of the BERTL-HIALCC approach with other methods [30].

Methods	$Accu_{y}$ (%)	$Prec_{n}$ (%)	$Reca_{l}$ (%)	$F_{score}$ (%)	CT (s)
BERTL-HIALCC	99.22	98.07	98.06	98.06	45
MPADL-LC3 Algorithm	99.09	98.01	97.20	97.07	52
mSRC Algorithm	88.21	85.21	91.78	86.78	64
Faster R-CNN Model	98.79	96.53	97.63	97.32	58
DAELGNN Model	98.73	97.95	96.39	96.80	55
RESNET-50	93.64	96.12	97.49	96.94	60
CNN Model	97.11	97.07	97.44	97.61	54
DL Algorithm	96.32	96.86	96.44	97.08	57

## Data Availability

The data presented in this study are available in this article.
